# Dual-energy spectral CT quantitative parameters for the differentiation of Glioma recurrence from treatment-related changes: a preliminary study

**DOI:** 10.1186/s12880-019-0406-5

**Published:** 2020-01-16

**Authors:** Yanchun Lv, Jian Zhou, Xiaofei Lv, Li Tian, Haoqiang He, Zhigang Liu, Yi Wu, Lujun Han, Meili Sun, Yadi Yang, Chengcheng Guo, Cong Li, Rong Zhang, Chuanmiao Xie, Yinsheng Chen, Zhongping Chen

**Affiliations:** 1 Department of Medical Imaging, Sun Yat-sen University Cancer Center, State Key Laboratory of Oncology in South China, Collaborative Innovation Center for Cancer Medicine, 651 Dongfeng Road East, Guangzhou, 510060 China; 2 Department of head and neck oncology, Phase 1 clinical trial ward, The cancer center of the fifth affiliated hospital of Sun Yat-sen University, Zhuhai, 519001 China; 3 Department of Radiology, Shantou Central Hospital, No.114 Waima Road, Shantou, 515041 Guangdong China; 4 Department of Neurosurgery/Neuro-oncology, Sun Yat-sen University Cancer Center, State Key Laboratory of Oncology in South China, Collaborative Innovation Center for Cancer Medicine, 651 Dongfeng Road East, Guangzhou, 510060 China

**Keywords:** Glioma, Dual energy spectral CT, Recurrence

## Abstract

**Background:**

Differentiating glioma recurrence from treatment-related changes can be challenging on conventional imaging. We evaluated the efficacy of quantitative parameters measured by dual-energy spectral computed tomographic (CT) for this differentiation.

**Methods:**

Twenty-eight patients were examined by dual-energy spectral CT. The effective and normalized atomic number (Z_eff_ and Z_eff-N,_ respectively); spectral Hounsfield unit curve (λ_HU_) slope; and iodine and normalized iodine concentration (IC and IC_N_, respectively) in the post-treatment enhanced areas were calculated. Pathological results or clinicoradiologic follow-up of ≥2 months were used for final diagnosis. Nonparametric and *t*-tests were used to compare quantitative parameters between glioma recurrence and treatment-related changes. Sensitivity, specificity, positive and negative predictive values (PPV and NPV, respectively), and accuracy were calculated using receiver operating characteristic (ROC) curves. Predictive probabilities were used to generate ROC curves to determine the diagnostic value.

**Results:**

Examination of pre-contrast λ_HU_, Z_eff_, Z_eff-N_, IC, IC_N_, and venous phase IC_N_ showed no significant differences in quantitative parameters (*P* > 0.05). Venous phase λ_HU_, Z_eff_, Z_eff-N_, and IC in glioma recurrence were higher than in treatment-related changes (*P* < 0.001). The optimal venous phase threshold was 1.03, 7.75, 1.04, and 2.85 mg/cm^3^, achieving 66.7, 91.7, 83.3, and 91.7% sensitivity; 100.0, 77.8, 88.9, and 77.8% specificity; 100.0, 73.3, 83.3, and 73.3% PPV; 81.8, 93.3, 88.9, and 93.3% NPV; and 86.7, 83.3, 86.7, and 83.3% accuracy, respectively. The respective areas under the curve (AUCs) were 0.912, 0.912, 0.931, and 0.910 in glioma recurrence and treatment-related changes.

**Conclusions:**

Glioma recurrence could be potentially differentiated from treatment-related changes based on quantitative values measured by dual-energy spectral CT imaging.

## Background

Differentiation between glioma recurrence and treatment-related changes (necrosis after operation or radiation, pseudoprogression after chemotherapy) remains a significant challenge. Clinically, the two entities have totally different consequences; however, both often share the same symptoms and show very similar features in conventional magnetic resonance imaging (MRI) and computed tomography (CT) [1, 2]. Given that the management strategies for tumor recurrence and treatment-related changes are completely distinct, it is crucial for clinicians to be able to differentiate these outcomes [3].

Many advanced imaging techniques such as functional magnetic resonance imaging (fMRI), positron emission tomography (PET), and single photon emission CT (SPECT) have been used in an attempt to distinguish these two conditions. These techniques, however, are imperfect, and accurate differentiation of treatment-related changes remains difficult [2–8].

In 2011, a novel spectral CT method known as gemstone spectral imaging (GSI) was introduced; GSI uses dual energy X-rays produced by the rapid switching of low (80 kVp) and high (140 kVp) tube voltages [9]. Quantitative parameters measured on GSI have been used to diagnose several tumor types [9–13].

Herein, we explored the use of quantitative parameters measured by dual-energy GSI-CT to differentiate between glioma recurrence and treatment-related changes.

## Methods

### Patients

The ethics committee at Sun Yat-sen University Cancer Center approved this retrospective study; all included patients provided informed consent. In all, 28 patients (13 men and 15 women; mean age: 39.3 ± 13.0 years) who underwent brain dual-energy GSI-CT were enrolled. All patients had undergone surgery for tumor removal, and the inclusion criteria were as follows: (1) histologically confirmed glioma; (2) the primary treatments were surgery, chemotherapy (temozolomide), or radiation therapy (total received dose: 40–60 Gy); and (3) detectable subsequently developed new contrast-enhanced lesions. Exclusion criteria were defined as definite contraindications for contrast-agent administration, cardiopathy, or pregnancy. The final diagnosis was determined based on either a second surgery or a follow-up examination. The follow-up evaluation was conducted at intervals of ≥2 months. In the case of follow-up diagnoses, treatment-related changes were confirmed in the event of complete disappearance of the enhancing lesion, partial resolution, if stable on subsequent follow-up images over a minimum period of 2 months, or if the patient was in a stable clinical state and showed no new neurologic symptoms. The glioma recurrence was based on the development of neurologic symptoms and a progressive increase in the size of the enhancing lesion or a new enhancing lesion on follow-up examination. Magnetic resonance imaging (MRI) enhancements or MR spectroscopy (MRS) were also used to help define treatment-related changes or glioma recurrence. All images were assessed in consensus by two radiologists (YL and JZ) with 20 and 8 years of experience in radiology, respectively.

### Dual energy gemstone spectral CT examination

The Discovery CT750HD scanner (GE Healthcare, Waukesha, WI, US) was used for scanning. The following scanning parameters in the GSI mode were used: tube voltage of 140 kV and 80 kV and 0.5-ms instantaneous switch; tube current, 0–600 mA automatic modulation; collimation thickness, 0.625 mm; rotation speed, 0.8 s; and helical pitch, 1.375. The total CT dose index volume used in this study was 18.28 mGy, 69.5% lower than the CT dose index volume of 59.89 mGy used for average conventional head scanning at our institution. An automated injector was used to inject an iodinated nonionic contrast agent (iopamidol 300; Bracco, Milan, Italy) at 2.8 mL/s and 1.5 mL/kg through the right ulnar vein. The scan’s venous phase delay time was 50 s.

### Acquisition of GSI quantitative parameters

**Fig. 1:**
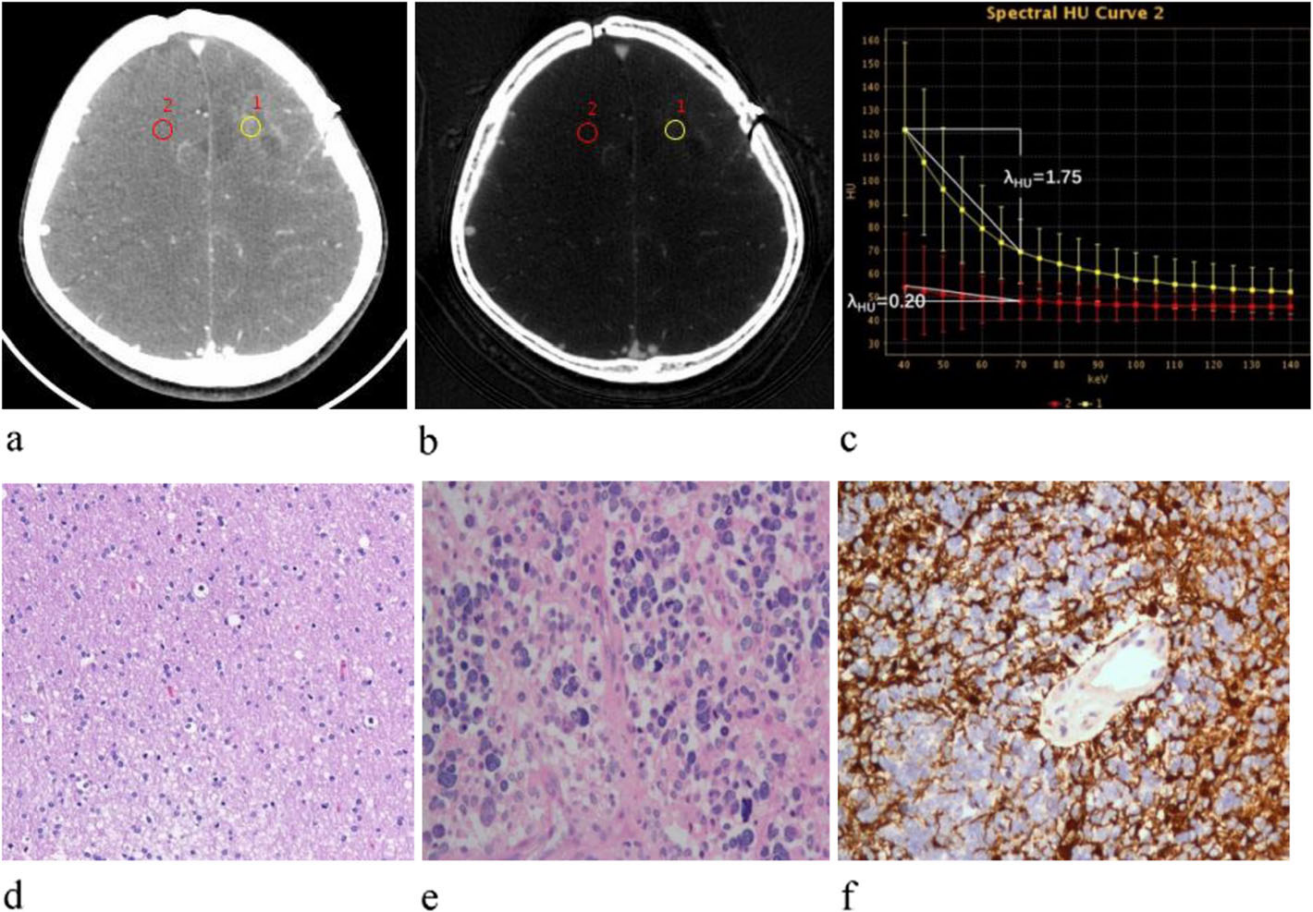
Contrast-enhanced venous phase GSI images show that IC and spectral curve were significantly different in glioma recurrence and the normal reference brain parenchyma. **a** Contrast-enhanced 70-keV monochromatic image (L1: area, 54.16 mm2; mean CT value, 69.33 HU; L2: 54.16 mm2; mean CT value, 48.06 HU). **b** Iodine-based materialdecomposition. image shows that IC in glioma recurrence and the normal reference brain parenchyma were 0.915 mg/cm3. and 0.113 mg/cm3 (L1: area, 54.16 mm2; mean IC, 9.15 · 100 μg/cm3; L2: area, 54.16 mm2; mean IC, 1.13 · 100 μg/cm3). **c** Graph shows spectral HU curve of glioma recurrence (yellow) and the normal reference brain parenchyma (red), slope of the curve representing glioma recurrence is much higher than the normal reference brain parenchyma (1.75 vs. 0.20). **d** The pathology noted after the first operation indicated astrocytoma (Grade II). **e** A large of tumor cells showed diffused distribution in the smear; eosinophil, nuclear were marked atypia, and the pathologic diagnosis was glioblastoma (Grade IV). **f** The GFAP was positive

**Fig. 2:**
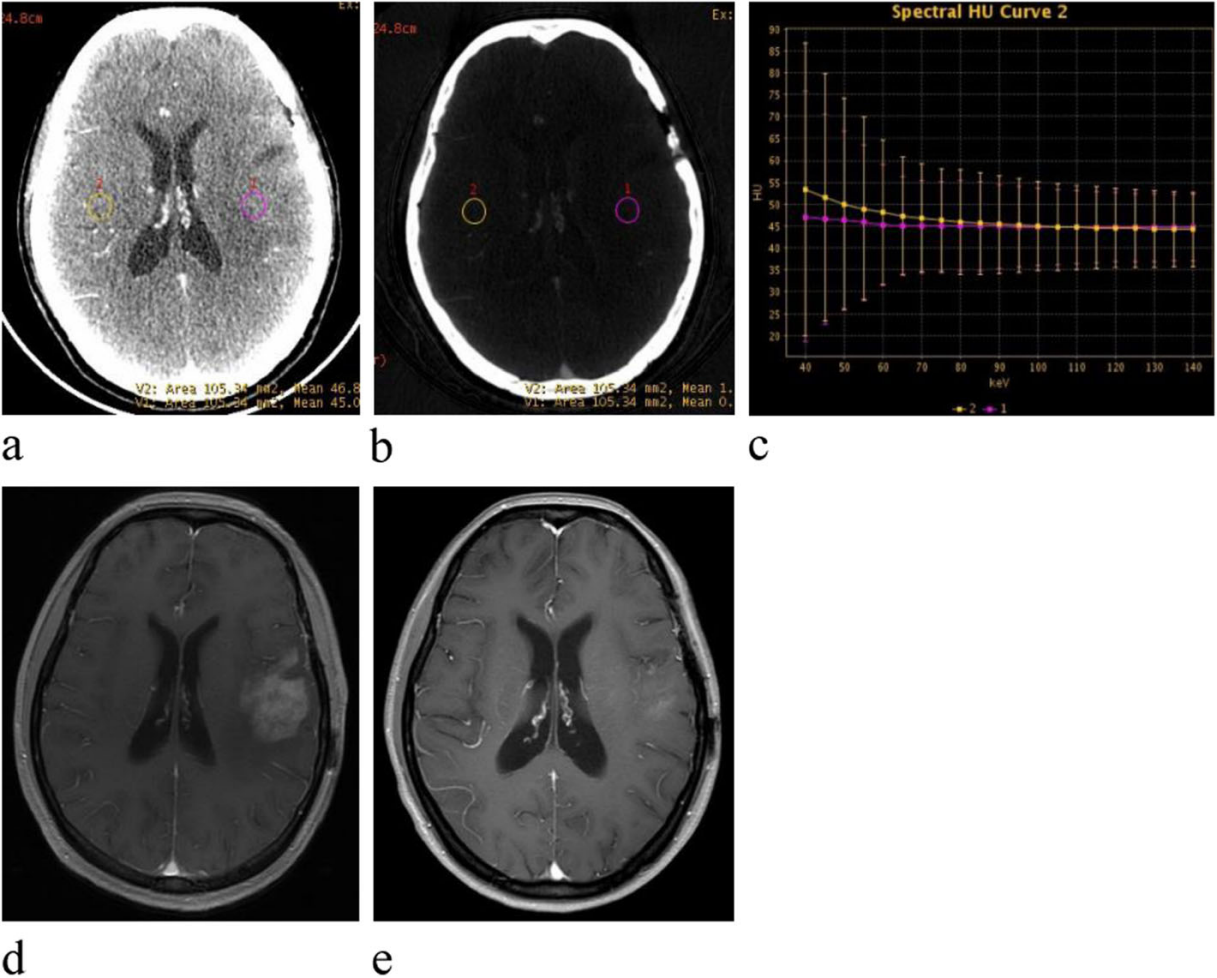
Contrast-enhanced venous phase GSI images show that IC and spectral curve were similar in treatment related necrosis and the normal reference brain parenchyma. **a** Contrast-enhanced 70-keV monochromatic image (L1: area, 105.34 mm2; mean CT value, 45.01 HU; L2: 105.34 mm2; mean CT value, 46.8 HU). **b** Iodine-based materialdecomposition image shows that IC in glioma recurrence and the normal reference brain parenchyma were 0.031 mg/cm3 and 0.122 mg/cm3 (L1: area, 105.34 mm2; mean IC, 0.31 · 100 μg/cm3; L2: area, 105.34 mm2; mean IC, 1.22 · 100 μg/cm3). **c** Graph shows spectral HU curve of glioma recurrence (violet) and the normal reference brain parenchyma yellow), slope of the curve representing glioma recurrence is similar with the normal reference brain parenchyma (0.07 vs. 0.22). **d** The same time with dual energy gemstone spectral CT scanning MRI T1WI enhanced image showed recurrence treatment related necrosis. **e** Seven months later, the MRI T1WI enhanced image showed the treatment related necrosis was obviously small with slight enhancement

The GSI viewer 4.5 (GE Healthcare) was used to acquire GSI images. The region of interest (ROI) was plotted on the pre-contrast scan and the reconstructed monochromatic venous phase data images on 70 keV. The ROI was targeted for most suspicious areas of tumor recurrence with nodular enhancement, with care to exclude calcification and minute vessel. The same ROI was copied on the other common brain parenchyma as a contrast. The CT-based effective atomic number (Z_eff_) and iodine concentration (IC) values in monochromatic images and iodine-based material-decomposition images for each ROI were automatically calculated (Figs. 1a, b and 2a, b). All ROIs were automatically copied on all monochromatic images and iodine-based material-decomposition images. All measurements were independently obtained by two radiologists.

### Data processing and statistical analysis

Z_eff_, IC (in mg/mL), and CT values on monochromatic images (40–140 keV) were calculated and exported by the average values of two radiologists. The Z_eff_ of the glioma (Z_eff-gli_) and IC of the glioma (IC_gli_) were normalized to values in the normal reference brain parenchyma (Z_eff-BP_ and IC_BP_) to obtain normalized Z_eff_ (Z_eff-N_) and IC (IC_N_): Z_eff-N_ = Z_eff-gli_/Z_eff-BP_ and IC_N_ = IC_gli_/IC_BP_, where BP is the normal reference brain parenchyma. The Hounsfield unit curve slope (λ_HU_) was indicated as the differences between the CT value on 40 keV and 70 keV divided by the energy difference (30 keV): λ_HU_ = (40 keV_HU_ − 70 keV_HU_)/30 keV (Fig. 1c and 2c).

Quantitative data were saved as means and standard deviation (^−^*x* ± *s*) or medians with interquartile range*.* All the GSI quantitative parameters were compared by two independent samples *t*-test and nonparametric tests. Predictive probabilities were used to generate ROC curves to evaluate the diagnostic value. Further, accuracy, positive predictive value (PPV), and negative predictive value (NPV) were calculated. The maximum Youden’s index value was chosen as the best threshold. Data were analyzed using statistical software package (SPSS version 21.0; SPSS Inc., IBM Corp, NY). *P* < 0.05 was considered to be statistically significant.

## Results

### Clinical and pathological results

In all, 28 patients were examined with dual energy gemstone spectral CT. Fifteen women [mean age, 36.9 ± 10.6 years] and 13 men [mean age, 42.2 ± 15.3 years] were included in the final analysis. A total of 30 lesions (12 glioma recurrence lesions, 18 treatment-related change lesions) were enrolled for evaluation.

The primary histopathology as per WHO 2007 classification was 15 Grade II (53.6%), 7 Grade III (25%), 6 Grade IV (21.4%). The primary histopathology was 6 glioblastomas (21.4%), 8 astrocytomas (28.6%), 3 anaplastic astrocytomas (10.7%), 2 oligodendrogliomas (7.1%), 3 anaplastic oligodendrogliomas (10.7%), 3 oligoastrocytomas (10.7%), 2 anaplastic oligoastrocytomas (7.1%), 1 ganglioglioma (3.6%). The primary treatments were 3 operation only (10.7%); 5 operation and radiation therapy (17.9%); 20 operation, radiation therapy, and chemotherapy (71.4%).

Pathology after operation showed glioma recurrence in 5 patients (5 lesions) and treatment-related changes in 2 patients (2 lesions). The recurrence group of second histopathology showed 2 glioblastomas (Grade IV), 1 astrocytoma (Grade II), 1 anaplastic oligodendroglioma (Grade III), 1 and anaplastic oligoastrocytoma (Grade III).

Six patients (7 lesions) without pathologic evaluation were finally classified into the glioma recurrence group up to a median period of 5 months (range, 2–24 months). Fifteen patients (16 lesions) without pathologic evaluation were finally classified into the treatment-related changes group up to a median period of 7.5 months (range, 2–46 months). Patient characteristics are listed in Table 1.

**Table 1:** Patient characteristics

Characteristic	Value
Age (mean years)	39.3 ± 13.0
Sex (No. of patients) (%)	
Male	13 (46.4)
Female	15 (53.6)
WHO classification (No. of lesions) (%)	
Grade II	15 (53.6)
Grade III	7 (25.0)
Grade IV	6 (21.4)
Primary treatment (No. of patients) (%)	
Operation	3 (10.7)
Operation + radiation therapy	5 (17.9)
Operation + radiation therapy + chemotherapy	20 (71.4)
Final diagnosis (No. of lesions) (%)	
Recurrence	11 (39.3)
Pathologic	5 (17.9)
Clinicoradiologic follow up	6 (21.4)
Treatment related changes	17 (60.7)
Pathologic	2 (7.1)
Clinicoradiologic follow up	15 (53.6)

### GSI quantitative parameters to differentiate between Glioma recurrence and treatment-related changes

**Fig. 3:**
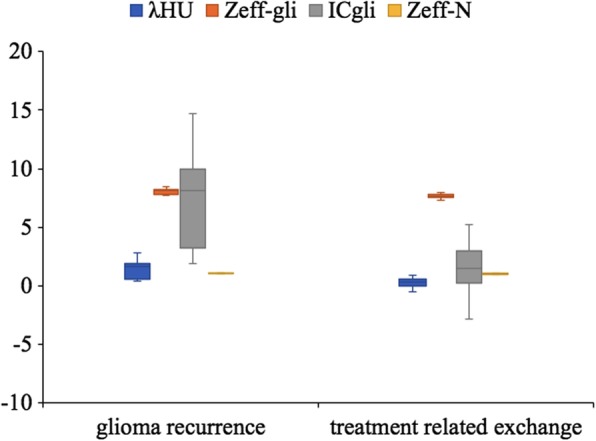
Box plots for glioma recurrence and treatment-related changes. The λHU, Zeff-gli, ICgli and Zeff-N measured in glioma recurrence were higher than in treatment-related changes in venous phase

**Fig. 4:**
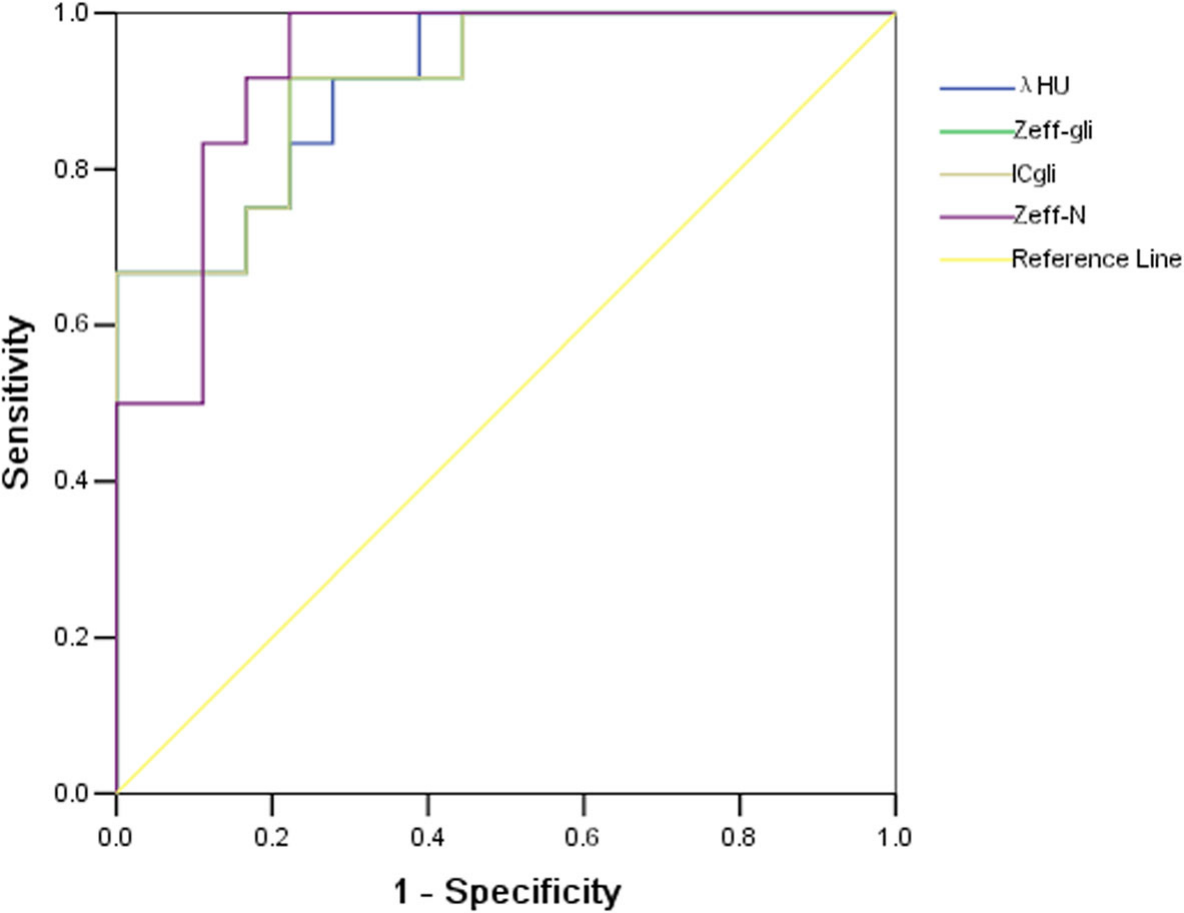
Graphs show receiver operating characteristic curves of λHU, Zeff-gli, ICgli and Zeff-N in venous phase for differentiating glioma recurrence from treatment-related changes in patients. The venous Zeff-N had the highest AUC (0.931), with the optimal threshold of 1.04 AUC = area under the curve

Table 2 enlists the differences in dual-energy spectral CT imaging quantitative parameters between glioma recurrence and treatment-related changes. Examination of pre-contrast λ_HU_, Z_eff_, Z_eff-N_, IC, IC_N_, and venous phase IC_N_ (*P* > 0.05) on dual-energy spectral CT images showed no significant differences in quantitative parameters. The mean λ_HU_ (*P* < 0.001) for glioma recurrence was 1.426 ± 0.762 vs. 0.314 ± 0.373 for treatment-related changes in the venous phase. In addition, the Z_eff_ (*P* < 0.001) for glioma recurrence was 8.034 ± 0.238 vs. 7.671 ± 0.151 for treatment-related changes in the venous phase. Similarly, the Z_eff-N_ (*P* < 0.001) for glioma recurrence was 1.058 ± 0.020 vs. 1.013 ± 0.024 for treatment-related changes. The IC (*P* < 0.001) for glioma recurrence was 7.319 ± 3.967 vs. 1.703 ± 2.049 for treatment-related changes in the venous phase (Fig. 3). The optimal venous phase λ_HU,_ Z_eff,_ Z_eff-N,_ and IC threshold was 1.03, 7.75, 1.04, and 2.85 mg/cm^3^, achieving a sensitivity of 66.7, 91.7, 83.3, and 91.7%; specificity of 100.0, 77.8, 88.9, and 77.8%; PPV of 100.0, 73.3, 83.3, and 73.3%; NPV of 81.8, 93.3, 88.9, and 93.3%; and accuracy of 86.7, 83.3, 86.7, and 83.3%, respectively (Table 3). The respective AUCs were 0.912, 0.912, 0.931, and 0.910 in glioma recurrence and treatment-related changes (Fig. 4).

**Table 2:** Difference of GSI quantitative parameters between glioma recurrence and treatment-related changes

GSI quantitative parameters	glioma recurrence	treatment related changes	*P* Value
Precontrast λHU	−0.007(− 0.477, 0.494)	−0.064(− 0.619, 0.310)	0.859
Precontrast Zeff	7.545 (7.353, 7.745)	7.520 (7.295, 7.653)	0.723
Precontrast Zeff-N	1.007 (0.996, 1.012)	1.005 (0.997, 1.012)	0.965
Precontrast IC	−0.108(−2.598, 2.649)	−0.375(− 3.33, 1.428)	0.790
Precontrast ICN	0.733 (0.509, 1.102)	0.969 (0.504, 1.086)	0.723
Venous phase λHU	1.426 ± 0.762	0.314 ± 0.373	< 0.001
Venous phase Zeff	8.034 ± 0.238	7.671 ± 0.151	< 0.001
Venous phase Zeff-N	1.058 ± 0.020	1.013 ± 0.024	< 0.001
Venous phase IC	7.319 ± 3.967	1.703 ± 2.049	< 0.001
Venous phase ICN	0.636(−2.140, 3.514)	0.827(− 0634, 1.740)	0.832

All *P* values for group comparisons were obtained by t test

Data in parentheses are medians with interquartile range

**Table 3:** GSI quantitative parameters for differential diagnosis of glioma recurrence and treatment-related changes

GSI Quantitative Parameters	AUC	Maximum Youden Index	Threshold of Parameters	Sensitivity (%)	Specificity (%)	PPV (%)	NPV (%)	Accuracy
Venous phase λHU	0.912 (0.812,1.012)	0.667	1.03	66.7 (56.7,76.7)	100.0 (90.0,110.0)	100.0 (90.0,110.0)	81.8 (71.8.91.8)	86.7 (76.7,96.7)
Venou phase Zeff	0.912 (0.810,1.014)	0.695	7.75	91.7 (81.5101.9)	77.8 (67.6,88.0)	73.3 (63.1,83.5)	93.3 (83.1103.5)	83.3 (73.1,93.5)
Venous phase Zeff-N	0.931 (0.843,1.019)	0.722	1.04	83.3 (74.5,92.1)	88.9 (80.1,97.7)	83.3 (74.5,92.1)	88.9 (80.1,97.7)	86.7 (77.9,95.5)
Venous phase IC	0.910 (0.810, 1.010)	0.695	2.85	91.7 (81.9101.5)	77.8 (68.0,87.6)	73.3 (63.5,83.1)	93.3 (83.5103.1)	83.3 (73.5,93.1)

*AUC* Area under the receiver operating characteristic curve

Data in parentheses are 95% confidence intervals (CIs)

## Discussion

A high incidence of treatment-related changes has been noted in patients who undergo post-operative radiotherapy or combined chemoradiotherapy with temozolomide. Moreover, routinely available CT and MRI techniques do not allow a reliable distinction between glioma recurrence and treatment-related changes [1, 14]. Moreover, the presence of a new contrast-enhanced lesion during follow-up imaging typically indicates a mixture of necrotic tissue and progressive tumor growth; this adds to the overall complexity of lesion characterization [3].

In this study, we used quantitative parameters measured on dual-energy spectral CT to differentiate between glioma recurrence and treatment-related changes. Additionally, the slope of λ_HU_, Z_eff_, Z_eff-N_, and IC in the venous phase was higher in patients with glioma recurrence than in those with treatment-related changes.

The λ_HU_ value was automatically generated for the given ROIs, describing the dynamic changes of measured CT Hounsfield units of ROIs against increasing keV values within the range of 40 to 140 keV [10]. In our study, we calculated λ_HU_ as the difference between the CT value on 40 keV and 70 keV divided by the energy difference (30 keV). Our results showed that the venous phase λ_HU_ in glioma recurrence was higher than in treatment-related changes, indicative of feasibility of enhancing venous phase λ_HU_ as a differentiating factor. The ROC analysis in our study revealed that the venous phase λ_HU_ was highly specific (100%) for differentiating glioma recurrence from treatment-related changes. These findings were similar to the findings in previous reports [10, 13]. Srinivasan et al. also reported that spectral HU curve is a potentially useful parameter to differentiate between benign and malignant neck pathologic findings [15].

Z_eff_ is also a quantitative index for characterization of composition of a nodule. Furthermore, it signifies the composite atom in a compound or mixture of various materials and is important in the prediction of X-rays’ interaction with a substance [10]. According to our study results, venous phase Z_eff_ and Z_eff-N_ were higher in glioma recurrence than in treatment-related changes, which was indicative of the feasibility of venous phase Z_eff_ and Z_eff-N_ as a differentiating factor; these results are consistent with the findings in previous reports [10, 13]. The results of our ROC analysis showed that the venous phase Z_eff_ was highly sensitive in differentiating glioma recurrence from treatment-related changes.

Lv et al. reported a linear relationship between the measured and actual iodine concentrations in their study upon testing tubes filled with known iodine concentrations and iodine concentrations measured from the iodine-based material-decomposition images [9]. Our study results showed that venous phase IC was higher in glioma recurrence than in treatment-related changes, thereby suggesting the potential of venous phase IC as a differentiating factor. The ROC analysis in our study revealed that the venous phase IC was highly sensitive for differentiating glioma recurrence from treatment-related changes. A previous report also suggested the usefulness of IC in thyroid nodules as a quantitative parameter to distinguish between malignant and benign nodules [10]. Furthermore, measured IC in lesions might be a useful quantitative parameter of the lesion’s blood supply [11, 12]. Moding et al. showed that dual energy CT is a powerful tool for monitoring vascular changes after radiation therapy [16]. Increased IC could also be attributed to changes in tumor-associated vascular patterns and an increased blood supply [17].

Our study showed no significant differences with respect to venous phase IC_N_, contradicted with venous phase IC. This may likely be because of the sample size being relatively small, and the fact that gliomas are a heterogeneous group of tumors, which sometimes showed up as poor soft tissue contrast on dual-energy spectral CT, leading to potential selection bias.

There are a few other limitations to this study. In our experience, the differential diagnosis of lesions in the vicinity of the skull base is rather challenging given the presence of many small blood vessels on the cerebral cortex; this might have led to inaccuracies in differential diagnosis. Second, it should be noted that all glioma-recurrence lesions in this study were not analyzed by biopsy; some were confirmed by follow-up evaluations. This may have influenced the study results. Third, relevant data on interobserver reliability are lacking, because images were assessed in consensus. Finally, tumor heterogeneity and spatial heterogeneity were not considered in this study. Hence, further large-scale prospective trials, with glioma classification and tumor heterogeneity are required to validate our results by dual-energy spectral imaging.

## Conclusions

Dual energy GSI-CT may potentially afford quantitative values to help differentiate between glioma recurrence and treatment-related changes. Thus, a dual-energy spectral CT would mean a second examination in addition to the routine MRI in clinical practice.

## Data Availability

The datasets generated and analyzed during the current study are available from the corresponding author on reasonable request.

## Abbreviations

GSI: gemstone spectral imaging
IC: iodine concentration
IC_N_: normalized iodine concentration
Z_eff_: effective atomic number
Z_eff-N_: normalized effective atomic number
λ_HU_: the slope of the spectral Hounsfield unit curve
